# The Effects of Bevacizumab in Augmenting Trabeculectomy for Glaucoma: A Systematic Review and Meta-Analysis of Randomized Controlled Trials

**DOI:** 10.1097/MD.0000000000003223

**Published:** 2016-04-18

**Authors:** Xiaoyan Liu, Liang Du, Ni Li

**Affiliations:** From the Department of Ophthalmology (XL, NL), West China Hospital, Sichuan University, Chengdu, Sichuan Province, China; and Chinese Evidence-Based Medicine/Cochrane Center (LD), Chengdu, Sichuan Province, China.

## Abstract

The aim of the study was to assess the effects of bevacizumab in augmenting trabeculectomy for glaucoma.

We searched the databases of Cochrane Library, PubMed, Embase, CNKI, and VIP. All the databases were retrieved from the time databases established to September, 2015. The keywords we used were as follows: “bevacizumab,” “anti-VEGF,” “avastin,” “trabeculectomy,” “glaucoma,” and so on. We used a method of the freedom word search and the MeSH search combined, which was recommended by Cochrane Systematic Review Manual 5.1.2. Randomized controlled trails (RCTs) of frequently used bevacizumab in trabeculectomy for glaucoma were included. Study selection, data extraction, quality assessment, and data analysis were performed according to the Cochrane standards.

Eight randomized controlled trails involving 212 eyes in the experimental (bevacizumab or bevacizumab + mitomycin C) groups and 214 eyes in the control (mitomycin C or placebo) groups were selected. Compared with placebo, bevacizumab significantly increased the complete success rate [OR = 2.79, 95%CI, (1.47, 5.29), *P* = 0.002], what else, bevacizumab also significantly decreased the intraocular pressure (IOP) [MD = 3.07, 95% CI, (0.87, 5.27), *P* = 0.006] at the 6-month after trabeculectomy and the number of antiglaucoma medications [MD = 1.23, 95% CI, (0.66, 1.80), *P* *<* 0.0001]. Additionally, it also increased the risk of bleb leak [OR = 5.24, 95% CI, (1.30, 21.10), *P* = 0.02]. When compared with mitomycin C (MMC), bevacizumab significantly increased the rate of encysted blebs [OR = 4.62, 95% CI, (1.02, 20.91), *P* = 0.05]. However, there was no significantly difference between the bevacizumab + MMC groups and MMC groups whatever the items were.

Bevacizumab was an effective way in trabeculectomy concerning the complete success rate, IOP, and anti-glaucoma medications reduction when compared with placebo; however, it increased the risk of bleb leakage. And it significantly increased the rate of encysted blebs compared with MMC.

## INTRODUCTION

Glaucoma as the second reason of blindness is a serious threat to human vision health in the world.^1^ Most commonly, the clinical course of open angle glaucoma is so insidious that the problem is found only when the visual function suffers serious damage. Thus, for open angle glaucoma, early diagnosis and treatment are very important. Usually, the operation indications of open angle glaucoma are uncontrolled cases with drugs, cases cannot tolerate medications. However, some researchers thought that once the diagnosis was clear, with significant disc and vision changes, filtration operations should be used as the preferred treatment.^2,3^ Trabeculectomy is the main technique of open angle glaucoma.^4^ There are, however, some limitations of the surgery. Scar formation and fibrosis in the process of wound healing may result in obstruction of filtration tract, leading to the operation failure.^5,6^ In recent 3 decades, due to the use of antimetabolites, such as MMC and 5-fluorouracil (5-FU), the rate of operation success has been higher than before.^7–10^ However, antimetabolites may bring some serious complications, such as low intraocular pressure, filtering bleb leakage, filtering bleb-associated endophthalmitis, epithelial toxicity, and so on.^11^ Hence, researchers have been searching for more effective and safer ways to inhibit scar formation and fibrosis. Recently, some researchers have found that bevacizumab may work in some ways.^12–14^

However, studies about this aspect were few, and high-quality researches were also seldom seen. Whether glaucoma patients after trabeculectomy could benefit more from bevacizumab than MMC or placebo, it has not been reviewed yet. The purpose of this study is to systemic review the efficacy and safety of bevacizumab in the trabeculectomy, providing more reliable evidences for clinical workers.

## METHODS

### Search Strategy

We searched the databases of Cochrane Library, PubMed, Embase, CNKI, and VIP. All the databases were retrieved from the time databases established to September, 2015. The keywords we used were as follows: “bevacizumab,” “anti-VEGF,” “avastin,” “trabeculectomy,” “glaucoma,” and so on. We used a method of the freedom word search and the MeSH search combined, which was recommended by Cochrane Systematic Review Manual 5.1.2. For a more comprehensive search, a manual search of cited references in published studies was done. Two researchers selected and assessed all included studies independently, and then cross-checked. Due to the fact that all analyses were based on previously published studies, the ethical approval was not necessary for our study.

### Data Extraction

Two researchers extracted study characteristics and outcome data independently. If there were some discrepancies, they would be resolved through discussion or a third researcher. Data that we collected were as follows: baseline characteristics, IOP, best-corrected visual acuity (BCVA), complete success rate (CS), quality success rate (QS), failure rate, the number of glaucoma medications, and adverse events.

### Statistical Analysis

Revman 5.0 (the Cochrane collaboration; http://www.cochrane.org/) was used for statistical analysis of the data. For continuous outcomes, mean difference (MD) or standard mean difference (SMD) with 95% confidence intervals (CI) was used to calculate the results; however, odds ratio (OR) with 95% confidence intervals (CI) was used for dichotomous outcomes. We used the chi-square test to assess heterogeneity between trials and the *I2* statistic to assess the extent of inconsistency. If there was a significant heterogeneity, a random-effect statistical model would be used to confirm the case results. A fixed-effect model for calculations of summary estimates was applied, unless there was a significant heterogeneity. Subgroup analysis was intended to explore clinical differences among trials.

## RESULTS

### Search Results

**FIGURE 1:**
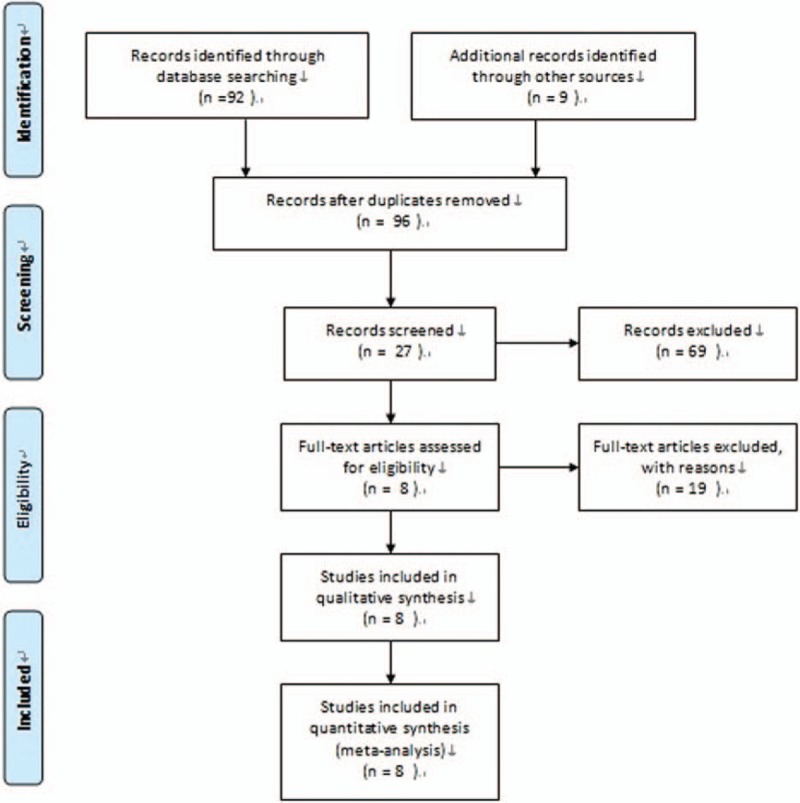
Flowchart showing systematic review search results.

We obtained 101 publications through searching literature databases and cited references. According to the inclusion criteria, only the RCTs for patients using bevacizumab during trabeculectomy were included. We eliminated the 74 articles by reading the title and abstract. Through further reading the full text, we ruled out 19 published papers, including 2 nonrandomized controlled trials, 7 retrospective case series, 6 retrospective controlled trials, and 4 prospective case series. Finally, we included 8 RCTs^15–22^ about use of bevacizumab in augmenting trabeculectomy for glaucoma in the meta-analysis. The process of literature screening was shown in Figure 1.

### Study Quality

**FIGURE 2:**
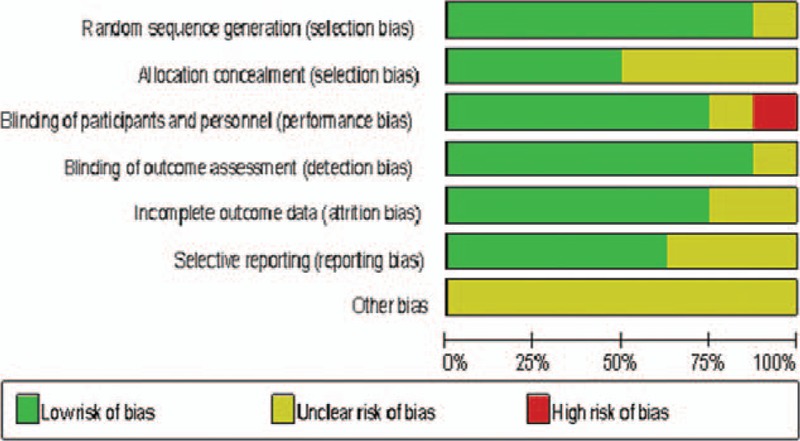
Quality evaluation of studies in the meta-analysis.

Table 1 described the specific information of the RCTs. A total of 426 eyes with 212 eyes in the experimental (bevacizumab or bevacizumab + MMC) groups and 214 eyes in the control groups separately were included in them. Figure 2 showed the methodological quality of the included RCTs, which was assessed by using the Cochrane Handbook 5.0.2. Seven studies^16–22^ of the included studies offered adequate descriptions of the randomization process. Five studies^16,17,19,21,22^ reported that masking was done either for the patients or for the practitioners; only 4 studies^16,18,21,22^ adequately stated allocation concealment. Six of included studies^15–18,21,22^ had stated incomplete outcome data. Furthermore, none of the papers adequately described other bias.

**TABLE 1:**
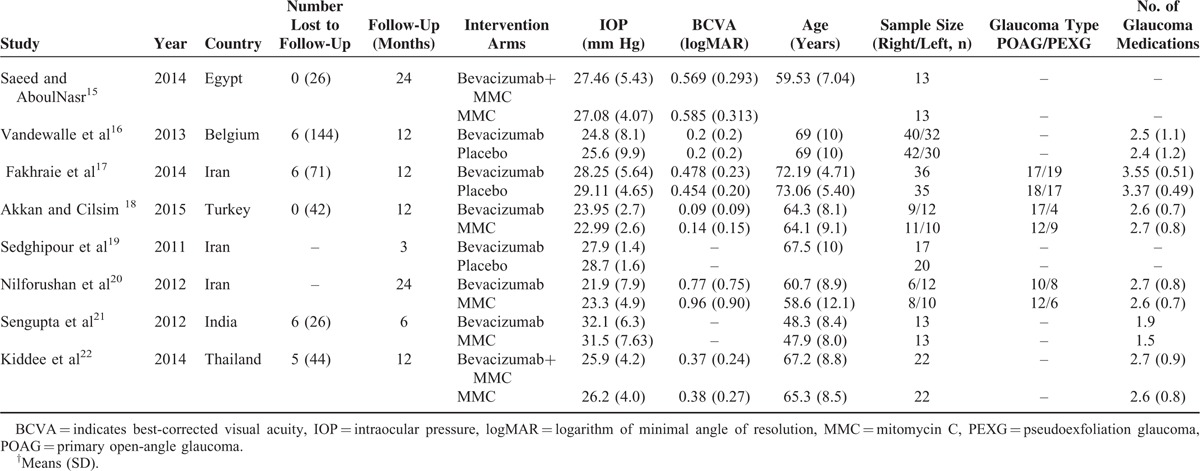
Baseline Characteristics of Included Studies^†^

### Studies and Baseline Characteristics

Characteristics of the 8 trials^15–22^ were shown in Table 1. Trabeculectomy were performed under local anaesthesia by experienced surgeons. All patients received bevacizumab, MMC, normal saline, and bevacizumab + MMC after trabeculectomy in the groups of bevacizumab, MMC, placebo, and bevacizumab + MMC respectively. Six trials reported antiglaucoma medications before trabeculectomy, and 3 studies reported the type of glaucoma including primary open-angle glaucoma (POAG) and pseudoexfoliation glaucoma (PEXG). The baseline characteristics of participants were displayed in Table 1. All studies were published between 2011 and 2015. Follow-up ranged from 3 to 24 months. There were comparable throughout age, IOP, BCVA, glaucoma medications, and glaucoma type in the papers.

### IOP

**FIGURE 3:**
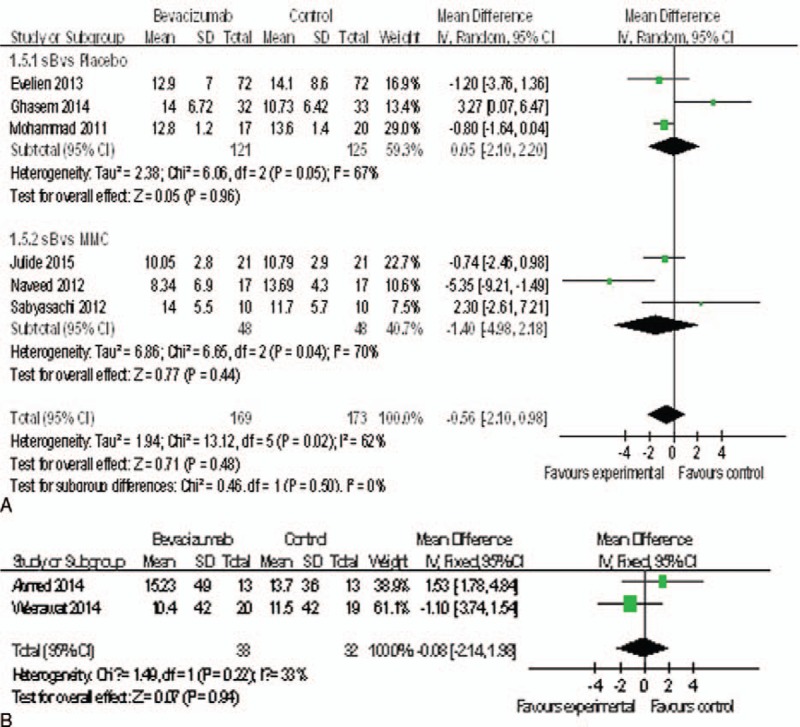
(A) Change of IOP at last month. (B) Change of IOP at last month. IOP = intraocular pressure.

**FIGURE 4:**
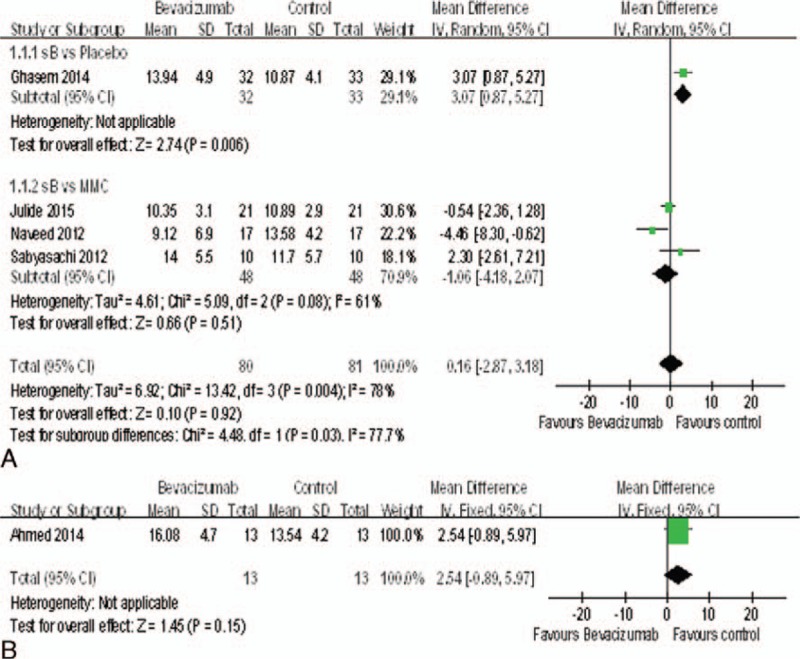
(A) Change of IOP at month 6. (B) Change of IOP at month 6. IOP = intraocular pressure.

All the studies^15–22^ reported IOP at last month. All study used the same scales to report IOP; thus the MD was used. Compared with bevacizumab groups, control groups including placebo groups (MD = 0.05, 95%CI, [−2.10, 2.20] *P* = 0.96) and MMC groups (MD = −1.40, 95%CI, [−4.98, 2.18] *P* = 0.44) were not associated with decreased IOP (Figure 3A). Additionally, bevacizumab+ MMC groups might have no advantage in decreasing IOP when compared with MMC groups (MD = −0.08, 95%CI, [−2.14, 1.98] *P* = 0.94) (Figure 3B). However, 5 studies^15,17,18,20,21^ reported IOP at the 6-month. The change of IOP in the bevacizumab groups was significantly higher than the placebo groups (MD = 3.07, 95%CI, [0.87, 5.27], *P* = 0.006). But there was no statistically significant difference between the bevacizumab groups and MMC groups (MD = −1.06, 95%CI, [−4.18, 2.07], *P* = 0.51) (Figure 4A), nor between the bevacizumab+ MMC groups and MMC groups (MD = 2.54, 95%CI, [−0.89, 5.97], *P* = 0.15) (Figure 4B).

### Complete Success Rate

**FIGURE 5:**
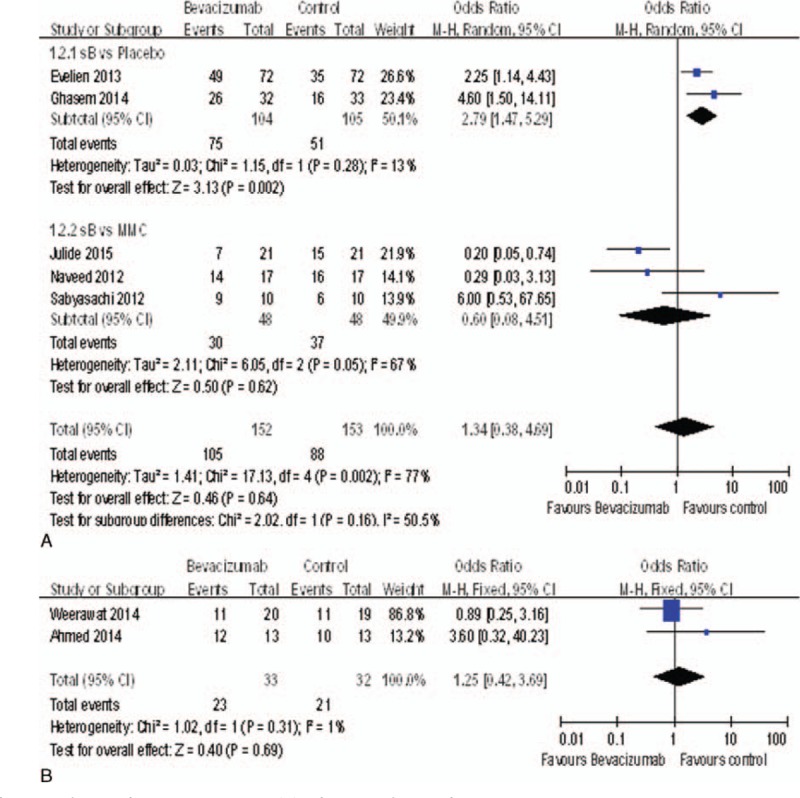
(A) Change of complete success rate. (B) Change of complete success rate.

Seven studies^15–18,20,21^ reported the complete success rate. The complete success rate of the bevacizumab groups was significantly higher than the placebo groups (OR = 2.79, 95%CI, [1.47, 5.29], *P* = 0.002). But there was no statistically significant difference between the bevacizumab groups and MMC groups (OR = 0.60, 95%CI, [0.08, 4.51], *P* = 0.62) (Figure 5A), nor between the bevacizumab+ MMC groups and MMC groups (OR = 1.25, 95%CI, [0.42, 3.69], *P* = 0.69) (Figure 5B).

### Failure Rate

**FIGURE 6:**
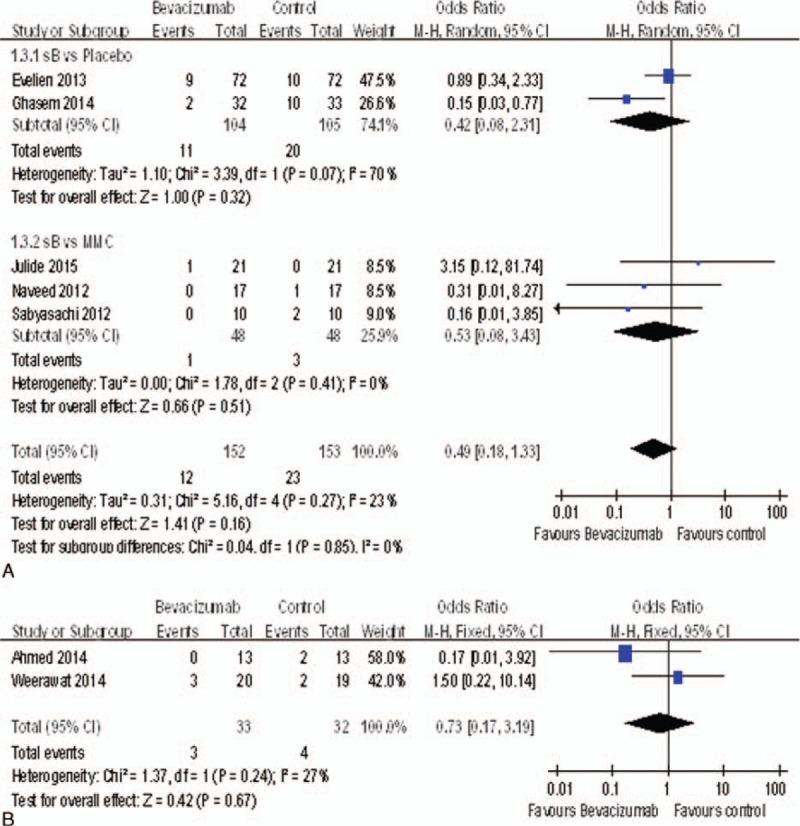
(A) Change of the failure rate. (B) Change of the failure rate.

Seven studies^15–18,20,21^ reported the failure rate. The failure rate of the bevacizumab groups was not significantly different with control groups including the placebo groups [OR = 0.42, 95%CI, (0.08, 2.31), *P* = 0.32] and MMC groups [OR = 0.53, 95%*CI*, (0.08, 3.43), *P* = 0.51] (Figure 6A). Otherwise, there was no significant difference between the bevacizumab + MMC groups and MMC groups [OR = 0.73, 95%*CI*, (0.17, 3.19), *P* = 0.67] (Figure 6B).

### BCVA

**FIGURE 7:**
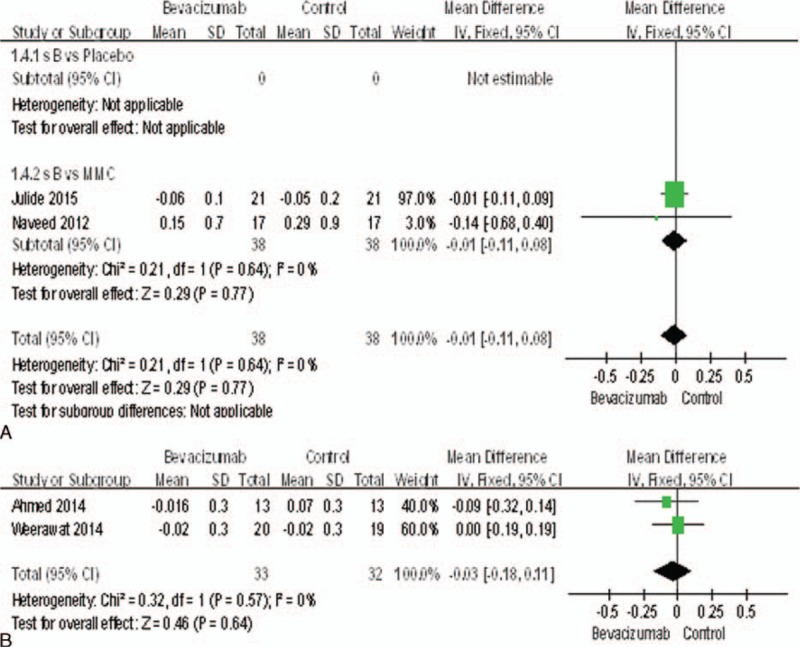
(A) Change of the BCVA. (B) Change of the BCVA. BCVA = best-corrected visual acuity.

Only 4 studies^15,18,20,22^ reported the BCVA. There was no statistically significant difference between bevacizumab and MMC groups (MD = −0.01, 95%CI, [−0.11, 0.08], *P* = 0.77) (Figure 7A), nor between the bevacizumab + MMC groups and MMC groups (MD = −0.03, 95%CI, [−0.18, 0.11], *P* = 0.64) (Figure 7B).

### Anti-Glaucoma Medications

**FIGURE 8:**
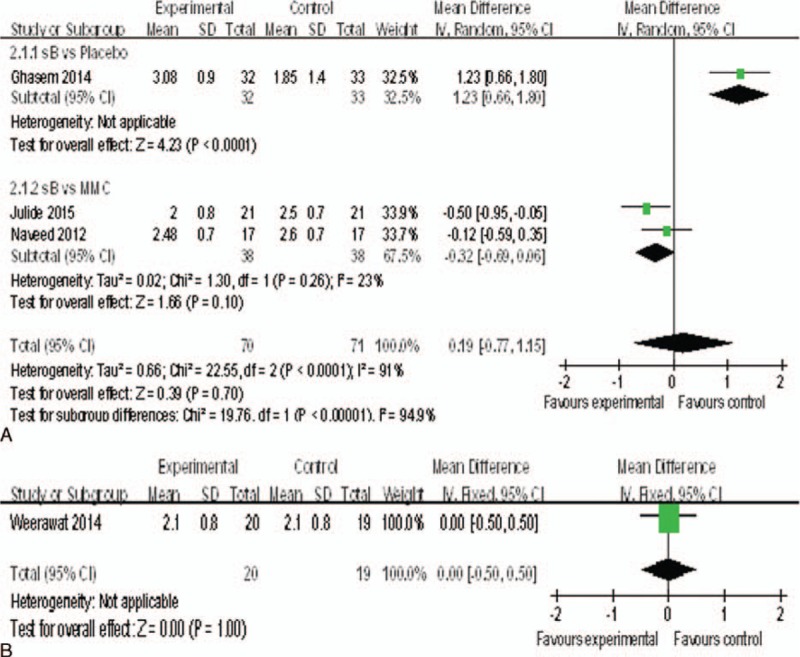
(A) Change of the antiglaucoma medications. (B) Change of the antiglaucoma medications.

Only 4 studies^17,18,20,22^ reported the change of antiglaucoma medications. There was statistically significant difference between bevacizumab and placebo groups (MD = 1.23, 95%CI, [0.66,1.80], *P* *<* 0.0001), but there was no statistically significant difference when compared with MMC groups (MD = −0.32, 95%CI, [−0.69,0.06], *P* = 0.10) (Figure 8A), nor between the bevacizumab+ MMC groups and MMC groups (MD = 0.00, 95%CI, [−0.50, 0.50], *P* = 1.00) (Figure 8B).

### Adverse Events

**FIGURE 9:**
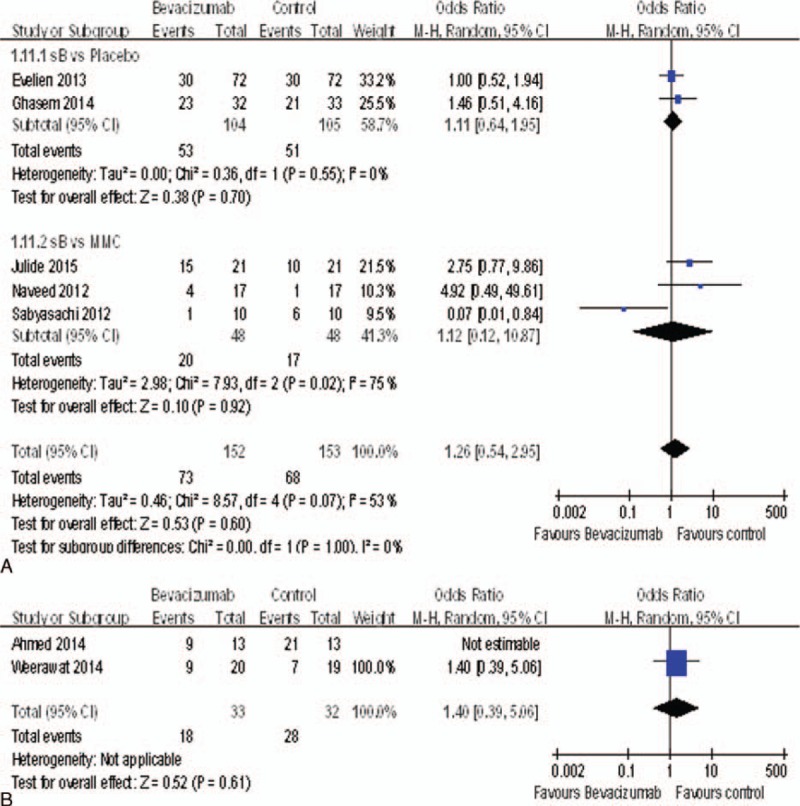
(A) Change of adverse events. (B) Change of adverse events.

We could analyze 7 studies^15–18,20–22^ for adverse events including bleb leak, hyphema, encysted blebs, anterior chamber shallowing, and so on. Fortunately, there was no statistically significant difference between bevacizumab and control groups, including the placebo groups (OR = 1.11, 95%CI, [0.64, 1.95], *P* = 0.70) and MMC groups (OR = 1.12, 95%CI, [0.12, 10.87] *P* = 0.92) (Figure 9A), nor between the bevacizumab + MMC groups and MMC groups (OR = 1.40, 95%CI, [0.39, 5.06], *P* = 0.61) (Figure 9B).

### Bleb Leak

**FIGURE 10:**
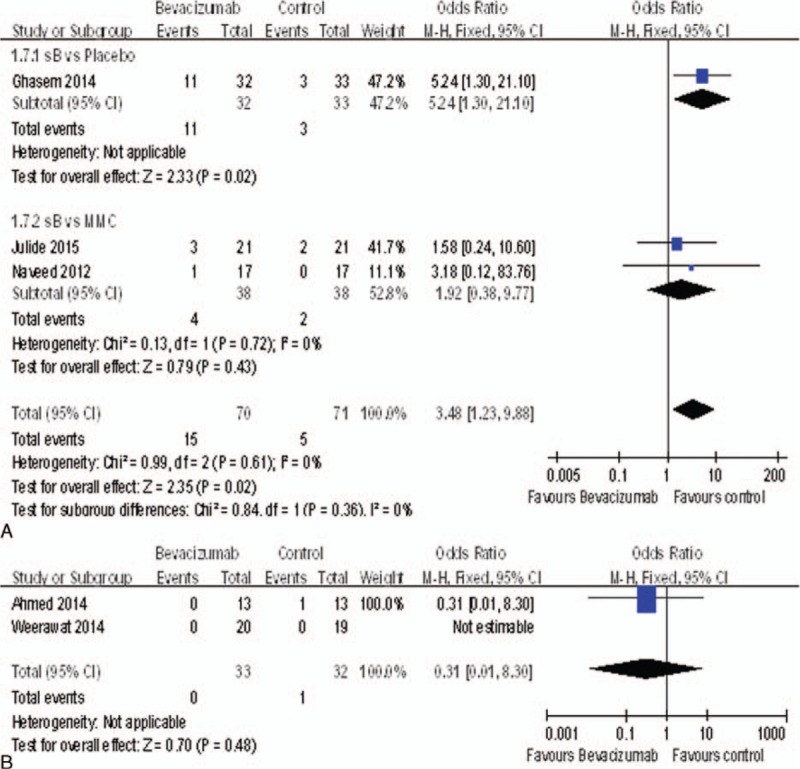
(A) The rate of bleb leak. (B) The rate of bleb leak.

We could analyze 5 studies^15,17,18,20,22^ for the bleb leak, and there was statistically significant difference between bevacizumab and placebo groups (OR = 5.24, 95%CI, [1.30, 21.10], *P* = 0.02). However, there was no statistically significant difference between bevacizumab and MMC groups (OR = 1.92, 95%CI, [0.38, 9.77], *P* = 0.43) (Figure 10A), nor between the bevacizumab + MMC groups and MMC groups (OR = 0.31, 95%CI, [0.01, 8.30], *P* = 0.48) (Figure 10B). Therefore, bevacizumab was associated with significantly increased the rate of bleb leak compared with placebo groups.

### Hyphema

**FIGURE 11:**
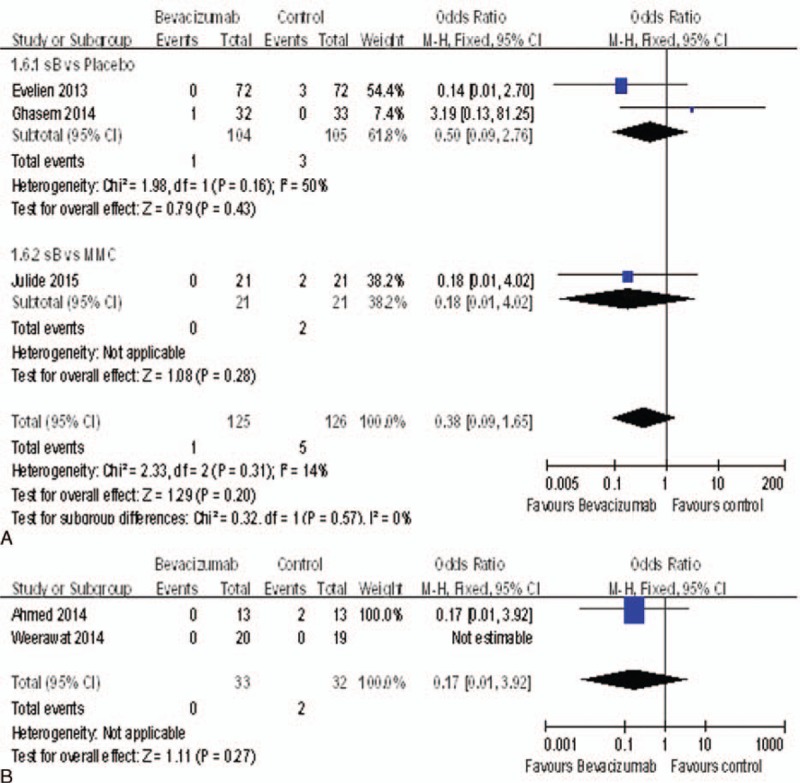
(A) The rate of hyphema. (B) The rate of hyphema.

There were 5 studies^15,16,18,20,22^ reported the rate of hyphema. There was no statistically significant difference between bevacizumab and control groups, including the placebo groups (OR = 0.50, 95%CI, [0.09, 2.76], *P* = 0.43) and MMC groups (OR = 0.18, 95%CI, [0.01, 4.02], *P* = 0.28) (Figure 11A), nor between the bevacizumab + MMC groups and MMC groups (OR = 0.17, 95%CI, [0.01, 3.92], *P* = 0.27) (Figure 11B).

### Encysted Blebs

**FIGURE 12:**
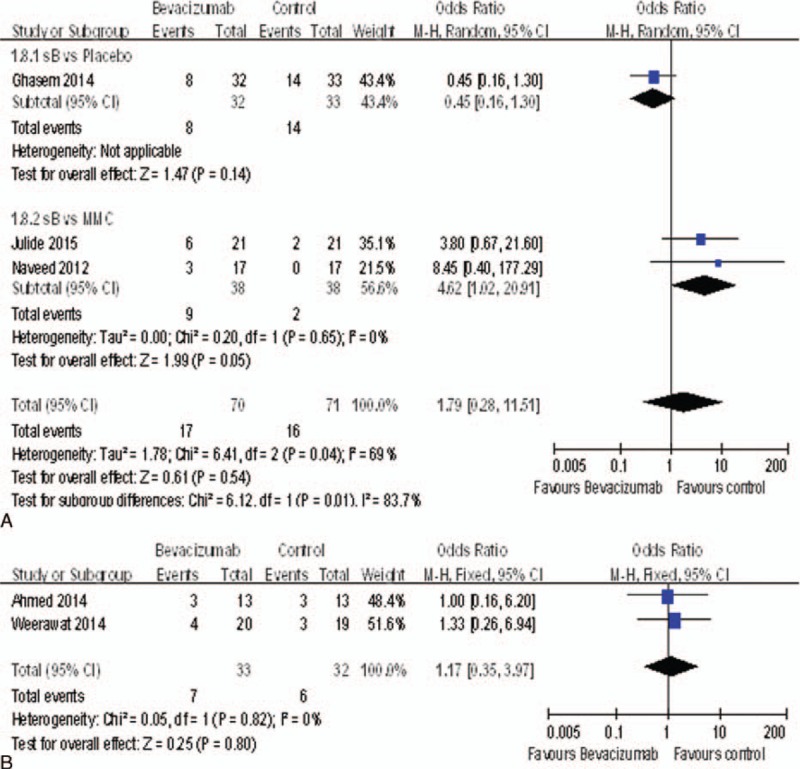
(A) The rate of encysted blebs. (B) The rate of encysted blebs.

There were 5 studies^15,17,18,20,22^ reported the rate of encysted blebs. The encysted blebs rate of the bevacizumab groups was significantly higher than the MMC groups (OR = 4.62, 95%CI, [1.02, 20.91], *P* = 0.05). But it was no statistically significant difference between the bevacizumab groups and the placebo groups (OR = 0.45, 95%CI, [0.16, 1.30], *P* = 0.14) (Figure 12A), nor between the bevacizumab + MMC groups and the MMC groups (OR = 1.17, 95%CI, [0.35, 3.97], *P* = 0.80) (Figure 12B). Therefore, bevacizumab was associated with significantly increased the rate of encysted blebs compared with MMC.

### Anterior Chamber Shallowing

**FIGURE 13:**
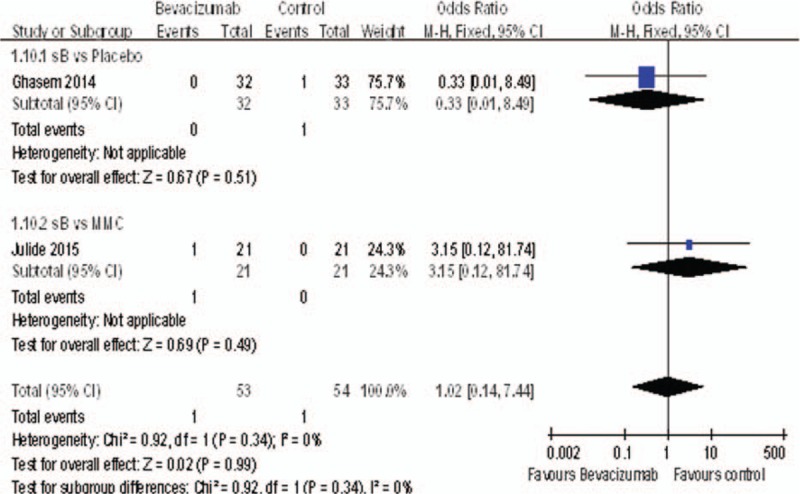
The rate of anterior chamber shallowing.

Only 2 studies^17,18^ reported the anterior chamber shallowing. There was no statistically significant difference between bevacizumab and control groups (OR = 1.02, 95%CI, [0.14, 7.44], *P* = 0.99) (Figure 13).

### Bleb Morphology

There were 5 studies^15,18,20–22^ reported bleb characteristics. Two^15,20^ showed no significantly difference between the experimental (bevacizumab or bevacizumab + MMC) groups and control groups. Two^21,22^ found that the vascularity scores of the experimental (bevacizumab or bevacizumab + MMC) groups were significantly lower when compared with the control groups at the 1-month follow-up. But these were not retained for longer time. One^18^ showed a statistically significant difference between 2 groups in regard to maximal bleb area, with the control group exhibiting more diffuse bleb area.

## DISCUSSION

The failure of trabeculectomy is mainly due to fibrosis and scar formation of subconjunctival tissue around the scleral flap and bleb during the wound-healing process.^23,24^ Bevacizumab, a humanized nonselective monoclonal antibody against vascular endothelial growth factor (VEGF), has been successfully used for diabetic retinopathy (DR),^25^ neovascular glaucoma,^26,27^ may work in some ways. As is known to all, tissue growth requires nutrients which provided by blood. Thus, bevacizumab are expected to act a role of inhibiting scar formation and fibrosis through the inhibition of angiogenesis information.^28^ On the other hand, the vascularization of conjunctiva is an important reason of bleb filtration failure. What is more, there were also evidences showed that VEGF had a direct effect on fibroblasts, which if inhibited by bevacizumab, scar formation, and fibrosis would be modulated.^28–32^ Previous studies found that the VEGF levels were elevated in patients who had a trabeculectomy.^33,34^ And the concentration of VEGF was significantly reduced after application of bevacizumab.^32,35^ Thus, bevacizumab may have the potential to work in trabeculectomy.

In the present study, 8 RCT studies were reviewed, consisting of 3 studies about bevacizumab vs placebo, 3 about bevacizumab vs MMC, and 2 about bevacizumab + MMC vs MMC. We found similar efficacy of reduction in the IOP and BCVA in the experimental (bevacizumab or bevacizumab + MMC) groups and control groups at last visit. Because of the lack of data reported in all phases of follow-up and trials with different durations, we chose the data from the end-point. The operative failure rate was also similar between the 2 groups. There were 5 studies, including 1 in the bevacizumab vs placebo groups, 3 in the bevacizumab vs MMC groups, and 1 in the bevacizumab + MMC vs MMC groups, reported IOP at the 6-month; we found the change of IOP was more remarkable in bevacizumab groups when compared with placebo groups (MD = 3.07, 95%CI, [0.87, 5.27], *P* = 0.006). However, there was no statistically difference when compared with MMC (MD = -1.06, 95%CI, [-4.18, 2.07], *P* = 0.51), nor between the bevacizumab + MMC groups and MMC groups (MD = 2.54, 95%CI, [-0.89, 5.97], *P* = 0.15). With respect to the complete success rate, bevacizumab was more likely to achieve complete success than placebo (OR = 2.79, 95%CI, [1.47, 5.29], *P* = 0.002), but there was no statistically significant difference between the bevacizumab groups and the MMC groups (OR = 0.60, 95%CI, [0.08, 4.51], *P* = 0.62), nor between the bevacizumab + MMC groups and MMC groups (OR = 1.25, 95%CI, [0.42, 3.69], *P* = 0.69). What is more, bevacizumab was associated with the reduction of antiglaucoma medications compared with placebo (MD = 1.23, 95%CI, [0.66,1.80], *P* *<* 0.0001).

For safety, results of adverse events were reported in 7 studies.^15–18,20–22^ Concerned overall adverse events, there was no statistically difference between the experimental (bevacizumab or bevacizumab + MMC) groups and control groups. And no one died patient was associated with bevacizumab and MMC in including studies. The adverse events included bleb leak, hyphema, encysted blebs, anterior chamber shallowing, hypotony, and so on. This meta-analysis showed bevacizumab not only increased the rate of bleb leak compared with placebo groups, but also increased the rate of encysted blebs compared with MMC.

Concerned with bleb morphology, 2 studies^21,22^ found bevacizumab had some advantages in reduce the vascularity scores in 1 month, which was similar to a recent cohort study.^36^ This might be associated with mechanism of bevacizumab, inhibiting the angiogenesis information. Akkan and Cilsim^17^ reported that the bevacizumab showed less efficiency in diffuse bleb area. This was in contrast with 1 recent study,^36^ revealing the bevacizumab group had greater extent.

Despite bevacizumab and MMC had similar efficacy in the IOP reduction and success rate, bevacizumab was much more expensive than MMC, with approximately $450 for each bevacizumab vial.^37^ If we use each vial of bevacizumab for multiple injections, the per dose price will potentially much lower than $450, depending on the number of injections per vial. However, each bevacizumab vial was allowed to use for only 1 injection because of the contamination outbreaks, discarding the leftover amount. Therefore, MMC might be the preferred choice concerned cost-effectiveness.

The present study is the meta-analysis that evaluates the efficiency and safety of bevacizumab in trabeculectomy. All the studies we included were RCT studies. Seven studies^16–22^ of the included studies offered adequate descriptions of the randomization process. The randomization process of 6 studies^16–18,20–22^ was generated by computer. Five studies^16,17,19,21,22^ reported that masking was done either for the patients or for the practitioners; only 4 studies^16,18,21,22^ adequately stated allocation concealment. Six of included studies^15–18,21,22^ had stated incomplete outcome data. Furthermore, none of the papers adequately described other bias.

Of course, there are some limitations in our meta-analysis that should be taken into consideration when considering the results. First, the number of RCTs and the sample sizes of these studies were very small, all of the studies^15–22^ enrolled only 426 eyes, resulting in the possibility of false-negative statistical error. Second, the varying definitions of surgical success in the literature and absence of patient's stratification into different types of glaucoma and risk of surgical failure should be taken into consideration. Furthermore, the different operative methods and procedures were performed by different surgeons would lead to an unavoidable potential bias. Additionally, the data came from the end-point owing to the lack of data reported in all phases of follow-up and trials with different durations introduced a potential heterogeneity. Finally, publication bias was inevitable.

## CONCLUSION

From the current evidences, we found bevacizumab was an effective way in trabeculectomy concerned the complete success rate, IOP, and antiglaucoma medications reduction when compare with placebo, but bevacizumab did not show any advantages when compared with MMC. However, bevacizumab not only increased the rate of bleb leak compared with placebo groups, but also increased the rate of encysted blebs compared with MMC. What is more, there was no difference between bevacizumab+ MMC and MMC whatever the items were. However, MMC might be the preferred choice concerned cost-effectiveness. Further intensive RCTs of large sample, high-quality, multiple centres, and vary phases of follow-up should be carried out to provide more clear and reliable evidence.

## References

[1] Foster PJ Johnson GJ Glaucoma in China: how big is the problem? *Br J Ophthalmol* 2001; 85:1277–1282. 11673287 10.1136/bjo.85.11.1277 PMC1723754

[2] Lavin MJ Wormald RPL Migdal CS The influence of prior therapy on the success of trabeculectomy. *Arch Ophthalmol* 1990; 108:1543–1548. 2244836 10.1001/archopht.1990.01070130045027

[3] Migdal C Gregory W Hitchings R Long-term functional outcome after early surgery compared with laser and medicine in open-angle glaucoma. *Ophthalmology* 1994; 101:1651–1656.discussion 1657. 7936562 10.1016/s0161-6420(94)31120-1

[4] Cairns JE Trabeculectomy. Preliminary report of a new method. *Am J Ophthalmol* 1968; 66:673–679. 4891876

[5] Nouri-Mahdavi K Brigatti L Weitzman M Outcomes of trabeculectomy for primary open-angle glaucoma. *Ophthalmology* 1995; 102:1760–1769. 9098275 10.1016/s0161-6420(95)30796-8

[6] Nguyen NX Kuchle M Martus P Quantification of blood–aqueous barrier breakdown after trabeculectomy: pseudoexfoliation versus primary open-angle glaucoma. *J Glaucoma* 1999; 8:18–23. 10084270

[7] Chen CW Huang HT Bair JS Trabeculectomy with simultaneous topical application of mitomycin-C in refractory glaucoma. *J Ocul Pharmacol* 1990; 6:175–182. 2127056 10.1089/jop.1990.6.175

[8] WuDunn D Cantor LB Palanca-Capistrano AM A prospective randomized trial comparing intraoperative 5-fluorouracil vs mitomycin C in primary trabeculectomy. *Am J Ophthalmol* 2002; 134:521–528. 12383808 10.1016/s0002-9394(02)01627-6

[9] Mostafaei A Augmenting trabeculectomy in glaucoma with subconjunctival mitomycin C versus subconjunctival 5-fluorouracil: a randomized clinical trial. *Clin Ophthalmol* 2011; 5:491–494. 21573097 10.2147/OPTH.S17328 PMC3090304

[10] Palanca-Capistrano AM Hall J Cantor LB Long-term outcomes of intraoperative 5-fluorouracil versus intraoperative mitomycin C in primary trabeculectomy surgery. *Ophthalmology* 2009; 116:185–190. 18930550 10.1016/j.ophtha.2008.08.009

[11] Anand N Arora S Clowes M Mitomycin C augmented glaucoma surgery: evolution of filtering bleb avascularity, transconjunctival oozing, and leaks. *Br J Ophthalmol* 2006; 90:175–180. 16424529 10.1136/bjo.2005.077800 PMC1860189

[12] Biteli LG Prata TS Subconjunctival bevacizumab as an adjuvant in first-time filtration surgery for patients with primary glaucomas. *Int Ophthalmol* 2013; 33:741–746. 23389840 10.1007/s10792-012-9704-4

[13] Simsek T Cankaya AB Elgin U Comparison of needle revision with subconjunctival bevacizumab and 5-fluorouracil injection of failed trabeculectomy blebs. *J Ocul Pharmacol Ther* 2012; 28:542–546. 22731246 10.1089/jop.2012.0035

[14] Suh W Kee C The effect of bevacizumab on the outcome of trabeculectomy with 5-fluorouracil. *J Ocul Pharmacol Ther* 2013; 29:646–651. 23621628 10.1089/jop.2012.0250

[15] Saeed AM AboulNasr TT Subconjunctival bevacizumab to augment trabeculectomy with mitomycin C in the management of failed glaucoma surgery. *Clin Ophthalmol* 2014; 8:1745–1755. 25246758 10.2147/OPTH.S67730 PMC4168860

[16] Vandewalle E Abegao Pinto L Van Bergen T Intracameral bevacizumab as an adjunct to trabeculectomy: a 1-year prospective, randomised study. *Br J Ophthalmol* 2014; 98:73–78. 24158846 10.1136/bjophthalmol-2013-303966

[17] Fakhraie G Ghadimi H Eslami Y Short-term results of trabeculectomy using adjunctive intracameral bevacizumab: a randomized controlled trial. *J Glaucoma* 2016; 25:182–188. 10.1097/IJG.0000000000000202 25493621

[18] Akkan JU Cilsim S Role of subconjunctival bevacizumab as an adjuvant to primary trabeculectomy: a prospective randomized comparative 1-year follow-up study. *J Glaucoma* 2015; 24:1–8. 23661044 10.1097/IJG.0b013e318287abf3

[19] Sedghipour MR Mostafaei A Taghavi Y Low-dose subconjunctival bevacizumab to augment trabeculectomy for glaucoma. *Clin Ophthalmol* 2011; 5:797–800. 21750613 10.2147/OPTH.S17896 PMC3130917

[20] Nilforushan N Yadgari M Kish SK Subconjunctival bevacizumab versus mitomycin C adjunctive to trabeculectomy. *Am J Ophthalmol* 2012; 153:352–357. 21982106 10.1016/j.ajo.2011.08.005

[21] Sengupta S Venkatesh R Ravindran RD Safety and efficacy of using off-label bevacizumab versus mitomycin C to prevent bleb failure in a single-site phacotrabeculectomy by a randomized controlled clinical trial. *J Glaucoma* 2012; 21:450–459. 21543993 10.1097/IJG.0b013e31821826b2

[22] Kiddee W Orapiriyakul L Kittigoonpaisan K Efficacy of adjunctive subconjunctival bevacizumab on the outcomes of primary trabeculectomy with mitomycin C: a prospective randomized placebo-controlled trial. *J Glaucoma* 2015; 24:600–606. 25393038 10.1097/IJG.0000000000000194 PMC4614532

[23] Hitchings RA Grierson I Clinicopathological correlation in eyes with failed fistulizing surgery. *Trans Ophthalmol Soc U K* 1983; 103 (Pt1):84–88. 6581641

[24] Addicks EM Quigley HA Green WR Histologic characteristics of filtering blebs in glaucomatous eyes. *Arch Ophthalmol* 1983; 101:795–798. 6847472 10.1001/archopht.1983.01040010795021

[25] Zhang ZH Liu HY Hernandez-Da Mota SE Vitrectomy with or without preoperative intravitreal bevacizumab for proliferative diabetic retinopathy: a meta-analysis of randomized controlled trials. *Am J Ophthalmol* 2013; 156:106–115. 23791371 10.1016/j.ajo.2013.02.008

[26] Zhou M Xu X Zhang X Clinical outcomes of Ahmed glaucoma valve implantation with or without intravitreal bevacizumab pretreatment for neovascular glaucoma: a systematic review and meta-analysis. *J Glaucoma* 2015; doi: 10.1097/j.0000000000000241. [Epub ahead of print]. 10.1097/IJG.0000000000000241 25719237

[27] Hwang HB Han JW Yim HB Beneficial effects of adjuvant intravitreal bevacizumab injection on outcomes of Ahmed glaucoma valve implantation in patients with neovascular glaucoma: systematic literature review. *J Ocul Pharmacol Ther* 2015; 31:198–203. 25714761 10.1089/jop.2014.0108

[28] Wong J Wang N Miller JW Modulation of human fibroblast activity by selected angiogenesis inhibitors. *Exp Eye Res* 1994; 58:439–451. 7523160 10.1006/exer.1994.1037

[29] Cheng G Xiang H Yang G Direct effects of bevacizumab on rat conjunctival fibroblast. *Cell Biochem Biophys* 2015; 73:45–50. 10.1007/s12013-015-0565-0 25656769

[30] O’Neill EC Qin Q Van Bergen NJ Antifibrotic activity of bevacizumab on human Tenon's fibroblasts in vitro. *Invest Ophthalmol Vis Sci* 2010; 51:6524–6532. 20574016 10.1167/iovs.10-5669

[31] Wilgus TA Ferreira AM Oberyszyn TM Regulation of scar formation by vascular endothelial growth factor. *Lab Invest* 2008; 88:579–590. 18427552 10.1038/labinvest.2008.36 PMC2810253

[32] Li Z Van Bergen T Van de Veire S Inhibition of vascular endothelial growth factor reduces scar formation after glaucoma filtration surgery. *Invest Ophtahlmol Vis Sci* 2009; 50:5217–5225. 10.1167/iovs.08-2662 19474408

[33] Seibold LK Sherwood MB Kahook MY Wound modulation after filtration surgery. *Surv Ophthalmol* 2012; 57:530–550. 23068975 10.1016/j.survophthal.2012.01.008

[34] Skuta GL Parrish RKII Wound healing in glaucoma filtering surgery. *Surv Ophthalmol* 1987; 32:149–170. 3328315 10.1016/0039-6257(87)90091-9

[35] Hu D-N Ritch R Liebmann J Vascular endothelial growth factor is increased in aqueous humor of glaucomatous eyes. *J Glaucoma* 2002; 11:406–410. 12362079 10.1097/00061198-200210000-00006

[36] Tai TY Moster MR Pro MJ Needle bleb revision with bevacizumab and mitomycin C compared with mitomycin C alone for failing filtration blebs. *J Glaucoma* 2015; 24:311–315. 25826644 10.1097/IJG.0b013e31829f9bd3

[37] Suzann Pershing Christine Pal Chee Asch Steven M Treating age-related macular degeneration: comparing the use of two drugs among medicare and veterans affairs populations. *Health Aff (Millwood)* 2015; 34:229–238. 25646102 10.1377/hlthaff.2014.1032

